# The Transcriptional Regulator Hbx1 Affects the Expression of Thousands of Genes in the Aflatoxin-Producing Fungus *Aspergillus flavus*

**DOI:** 10.1534/g3.118.200870

**Published:** 2018-11-13

**Authors:** Jeffrey W. Cary, Sarah Entwistle, Timothy Satterlee, Brian M. Mack, Matthew K. Gilbert, Perng K. Chang, Leslie Scharfenstein, Yanbin Yin, Ana M. Calvo

**Affiliations:** * Food and Feed Safety Research Unit, USDA/ARS, Southern Regional Research Center, New Orleans, Louisiana; † Department of Biological Sciences, Northern Illinois University, DeKalb, Illinois

**Keywords:** *Aspergillus flavus*, *hbx1*, secondary metabolism, fungal development, transcriptome

## Abstract

In filamentous fungi, homeobox proteins are conserved transcriptional regulators described to control conidiogenesis and fruiting body formation. Eight homeobox (*hbx*) genes are found in the genome of the aflatoxin-producing ascomycete, *Aspergillus flavus*. While loss-of-function of seven of the eight genes had little to no effect on fungal growth and development, disruption of *hbx1*, resulted in aconidial colonies and lack of sclerotial production. Furthermore, the *hbx1* mutant was unable to produce aflatoxins B_1_ and B_2_, cyclopiazonic acid and aflatrem. In the present study, *hbx1* transcriptome analysis revealed that *hbx1* has a broad effect on *A. flavus* gene expression, and the effect of *hbx1* increases overtime, impacting more than five thousand protein-coding genes. Among the affected genes, those in the category of secondary metabolism (SM), followed by that of cellular transport, were the most affected. Specifically, regarding the effect of *hbx1* on SM, we found that genes in 44 SM gene clusters where upregulated while 49 were downregulated in the absence of *hbx1*, including genes in the SM clusters responsible for the synthesis of asparasone, piperazine and aflavarin, all known to be associated with sclerotia. In addition, our study revealed that *hbx1* affects the expression of other transcription factor genes involved in development, including the conidiation central regulatory pathway and *flb* genes.

The opportunistic phytopathogen, *Aspergillus flavus*, is often found colonizing oil seed crops such as peanut, corn, sorghum, tree nuts and cotton (Robens and Cardwell 2003). Dispersal of this fungus proceeds rapidly in the field through production of asexual spores termed conidia present on specialized structures denominated conidiophores. Once the fungus has colonized the crop it can survive in the field under harsh conditions for several years by forming resistant structures termed sclerotia (Horn *et al.*, 2014). Upon colonization of the plant, *A. flavus* produces a number of mycotoxins, including the highly carcinogenic family of toxins known as aflatoxins (Bhatnagar *et al.*, 2018). Contaminated crops are often destroyed or significantly reduced in value leading to substantial economic losses in the range of one billion US dollars annually during years of severe aflatoxin outbreaks (Robens & Cardwell 2003, & Wu *et al.* 2008). In developing nations where legislation is often not in place to regulate the allowable levels of aflatoxins in susceptible crops, consumption of aflatoxin-contaminated food can lead to immunosuppression, liver cancer, and in some cases death (Yard *et al.* 2013).

Successful control of aflatoxin contamination in crops will depend in part on research efforts directed toward understanding the regulatory mechanisms controlling *A. flavus* dissemination and survival, as well as mycotoxin biosynthesis and pathogenicity. It has been shown that *A. flavus* development is genetically linked to secondary metabolism, including the production of mycotoxins (Calvo and Cary 2015). Among several important regulators of fungal development and secondary metabolism is the light-responsive global regulator VeA. This fungal-specific protein has been shown to regulate asexual and sexual development as well as production multiple secondary metabolites in many fungal genera (Calvo *et al.*, 2016), including *Aspergillus* (Kato *et al.*, 2003, Duran *et al.* 2007, Dhingra *et al.* 2013, Lind *et al.* 2015). In *A. flavus*, loss of VeA results in increased conidiation, absence of sclerotia, and suppression of secondary metabolite production including aflatoxin, aflatrem, and cyclopiazonic acid (Duran *et al.* 2007). In addition to VeA, the arginine methyltransferase RmtA has been shown to be a positive regulator of both aflatoxin production and asexual development (Satterlee *et al.* 2016). Another example is RtfA, a homolog of a putative member of the *Saccharomyces cerevisiae paf1* complex (Warner *et al.*, 2007), that is also required for normal aflatoxin biosynthesis, sclerotial production and conidiation (Lohmar *et al.* 2016). Other examples are the genes encoding transcription factors *mtfA* (Zhuang *et al.* 2016) *nsdC*, and *nsdD* (Cary *et al.* 2012). The global regulator MtfA is a negative regulator of conidiation, required for normal maturation of sclerotia, and a positive regulator of aflatoxin production (Zhuang *et al.* 2016). Both *nsdC* and *nsdD* also demonstrated a role in the regulation of conidiophore development, and are essential for sclerotial formation, as well as influencing production of aflatoxin (Cary *et al.* 2012).

Recently homeobox domain transcription factor genes were identified in *A. flavus*, and disruption of the homeobox 1 (*hbx1*) gene abolished production of conidia and sclerotia as well as production of several mycotoxins (Cary *et al.* 2017). The *hbx1* gene was also shown to regulate expression of several development regulators such as *brlA* (Cary *et al.*, 2012), a keystone in the induction of conidiation (Adams *et al.* 1998). Alongside the effect on developmental regulators, expression of the aflatoxin specific transcription factor *aflR* and the global regulator *veA* were altered in the absence of *hbx1* possibly contributing to the observed decrease in the production of several mycotoxins such as aflatrem, cyclopiazonic acid, and aflatoxin (Cary *et al.*, 2017).

Based on the profound effect that *hbx1* has on development and secondary metabolism in *A. flavus*, this gene represents a potential target for new strategies to control aflatoxin contamination of food and feed crops by *A. flavus*. To gain further insight into the regulatory scope of *hbx1* we performed a transcriptome analysis. The impact of *hbx1* on the gene expression profile of *A. flavus* was assessed over three-time points. Several thousand genes were under *hbx1* control indicating that *hbx1* is a global regulator, and its influence increased with time. An elevated number of transcription factors and developmental regulators were shown to be *hbx1*-dependent. Furthermore, a large numbers of secondary metabolite gene clusters are also affected by *hbx1*, among them seven are associated with known metabolites.

## Materials & Methods

### Strains used and growth conditions

*Aspergillus flavus* strains used in this study were the AF70 control, AF70 Δ*hbx1* and a genetically complemented Δ*hbx1* mutant (designated AF70 Δ*hbx1*-COM) as described in Cary *et al.* (2017). Strains were point inoculated onto double strength 5/2 agar (50 mL V8 juice, 40 g agar, pH 5.2 per liter of medium (Chang *et al.* 1993) supplemented with 3.0 g ammonium sulfate and 1 mg/ml uracil (termed 2X V8 ASU) and incubated in the light at 30° for 6 days to promote conidiation. Conidia were collected from plates in 0.01% Triton X-100 and stored at 4°. Due to the inability of the Δ*hbx1* mutant to conidiate, cultures were maintained at -80° as glycerol stocks containing agar plugs of fungal mycelia.

### Sequence analysis of plant homologs

Using the amino acid sequence for Hbx1 as query (XP_002380469.1) a BLASTp search was performed to identify possible homologs of Hbx1 in selected plant species. Species and sequences used were *Arachis hypogaea* (AKN10291.1), *Zea mays* (NP_001140916.1), *Gossypium arboretum* (XP_017643272.1) and *Arabidopsis thaliana* (AAA56907.1). A MAFFT multiple sequences alignment (https://mafft.cbrc.jp/alignment/software/) was performed to align the sequences and visualized using BoxShade (https://embnet.vital-it.ch/software/BOX_form.html).

### RNA sequencing study

#### RNA preparation and sequencing:

Inoculated approximately 5 × 10^5^ conidia/ml of the AF70 control and the Δ*hbx1*-COM mutant into 500 ml peptone minimal salts (PMS; not conducive to aflatoxin production) (Buchanan and Lewis 1984) broth supplemented with 1 mg/ml uracil (PMSU) in 1 liter Ehrlenmeyer baffle flasks. Cultures were incubated at 30° in the dark with shaking at 250 rpm for 24 h. Mycelia of the AF70 Δ*hbx1* mutant were scraped from the surface of four 2X V8 ASU top agarose (0.5% agarose I, Amresco, Solon, OH) plates and placed in 25 mL 2X V8 ASU broth in a 50 ml Sarsteadt tube. Equal amounts of mycelia were macerated for 10 sec using a tissue grinder (Tissumizer SDT1810, Tekmar, Cincinnati, OH) then transferred into 500 ml PMSU broth in 1 liter Ehrlenmeyer baffle flask. Incubated at 30° in the dark with shaking at 250 rpm for 24 h. Collected mycelia from cultures of each of the three strains by filtering through sterile miracloth, transferred 0.5g wet weight into 25 ml of PDBU broth in 250 ml Ehrlenmeyer flasks (4 replicates) and incubated statically in the dark for 6 h (time point for initiation of aflatoxin gene expression), 24h and 48 h (approximate time points for initiation of conidia and sclerotia production, respectively). Cultures were filtered through sterile miracloth and the fungal tissue collected, frozen in liquid nitrogen and stored at -80°. The frozen mycelial samples were ground under liquid nitrogen with motar and pestle until powdered and transferred to a 50ml Sarstedt tube and stored frozen at -80 until ready for RNA extraction. RNA was isolated from 100-200 mg of the frozen ground mycelial samples using the TRI Reagent (Sigma T9424-100ML) and following the standard Direct-zol RNA MiniPrep kit (ZYMO Research, Irvine, CA) protocol using the double washes modification. RNA quality and quantity were determined using the Experion Automated Electrophoresis Station (Bio-Rad). Frozen RNA samples were shipped overnight on dry ice to North Carolina State University’s Genomics Sciences Laboratory for RNA sequencing. RNA libraries were prepared using the Ultra Directional RNA library prep kit from NEB using the manufacturer’s protocol for NEBNext PolyA mRNA magnetic isolation module. Sequencing was carried out by Illumina HiSeq 2500 at 125 bp single end reads.

#### RNA data analysis:

##### Read mapping

The single-end reads of three strains (Control, Δhbx1, Δhbx1-COM) each with three replicates at three time points (6 h, 24 h, and 48 h) were separately aligned to the reference genome (Nierman *et al.*, 2015) using HISAT2 (Kim *et al.*, 2015) version 2.0.5. The command used was hisat2 -x reference_genome_index –U fastq_file -S output_file.sam. HISAT2 utilizes Bowtie2 (Langmead & Salzberg 2012) and was run using software version 2.3.

##### Read counts

The mapped reads in SAM format were then analyzed using the feature Counts tool from the Subread package (Liao *et al.*, 2013) version 1.6.0. This tool was employed to return a table of read counts for each gene. The command used was featureCounts -a reference_genome.gtf -p -s 2 -o output_file –primary input_file.sam. A bash script was used to combine all the separate read count files into one table.

##### Differentially expressed coding genes (DEGs)

The table of read counts was used as input for the R limma package (Ritchie *et al.*, 2015). This package was used to determine DEGs by comparing read counts between two strains: Control *vs.* Δhbx1 and Control *vs.* Δhbx1-COM. These comparisons were made at all three-time points: 6 h, 24 h, 48 h. The replicates of each condition at each time point were combined during this step of the analysis. The RPKM function in the R edgeR package (Robinson *et al.*, 2009) determined the reads per kilobase per million (RPKM) values for all the genes.

Bash and Perl scripts were developed to parse the DEGs and RPKM data. An Excel file was created with the RPKM values for all genes across all conditions. FungiFun2 (Priebe *et al.*, 2014) was used for FunCat term annotation of DEGs from Control *vs.* Δhbx1 and Control *vs.* Δhbx1-COM. FungiDB (Basenko *et al.*, 2018) was used for GO term annotation.

##### Functional annotation and generation of gene lists

Secondary Metabolite gene clusters (SMGs) were extracted from Ehrlich and Mack (2014). In addition, a list of transcription factors (TFs) were derived from the Fungal Transcription Factor Database (http://ftfd.snu.ac.kr/intro.php) (Park *et al.* 2008) for *A. flavus* and mapped to differentially expressed genes in *A. flavus*. Functional annotations of these transcription factors were obtained from NCBI. R (R Core Team 2017) version 3.4.1, specifically the ggplot2 package (Wickham 2009), was used to make statistical figures. The Venn diagrams were made using the R package VennDiagram (Chen & Boutros 2011). Fungal development-related genes from *Aspergillus* species were reviewed in Table S2 from Krijgsheld *et al.* (2013). FASTA sequences of these genes were used to search against the *A. flavus* genome to identify developmental genes.

The list of DEGs from the study performed by Dolezal and collaborators (Dolezal *et al.* 2013) was compared to the *hbx1* DEGs to search for potentially *hbx1*–dependent virulence genes. Furthermore, we specifically looked for virulence-related secretory genes. The list of possible virulence-related *hbx1* genes was compared to the *A. flavus* secretome-related genes in the FunSecKB2 database (Meinken *et al.*, 2014). For higher confidence in results only the list of “curated secreted” and “highly likely secreted” genes in FunSecK2 were used.

##### Weighted gene network co-expression analysis

The gene co-expression network was made using WGCNA (Weighted Gene Network Co-expression Analysis) with a signed network, the biweight mid-correlation method, and a soft-thresholding power of 9. Variance stabilized counts from DESeq2 were used as input to WGNCA (Langfelder and Horvath 2008). Genes with missing values or zero variance were filtered out using the goodSamplesGenes function within the WGCNA package. Visualization of gene networks using wild-type *A. flavus* data were shown using Cytoscape v3.6.0 with the Edge-weighted Spring Embedded layout with minor manual adjustment. Relative edge weight values were calculated for the entire module containing *hbx*, and First Neighbor nodes were selected for additional analysis.

### Data availability

Table S1 contains calculated expression values of sequenced RNA samples along with corresponding p-values. Table S2 contains a selected list of fungal developmental regulators that are shown to be *hbx1*-dependent. Table S3 is a subset of Table S1 that shows all known transcription factors in *A. flavus* and their corresponding expression pattern in regard to presence or absence of *hbx1*. The data are publicly available at NCBI’s SRA repository with the SRA Accession #: PRJNA494425. Supplemental material available at Figshare: https://doi.org/10.25387/g3.7304252.

## Results

### Hbx1 is not conserved in plants species

**Figure 1:**
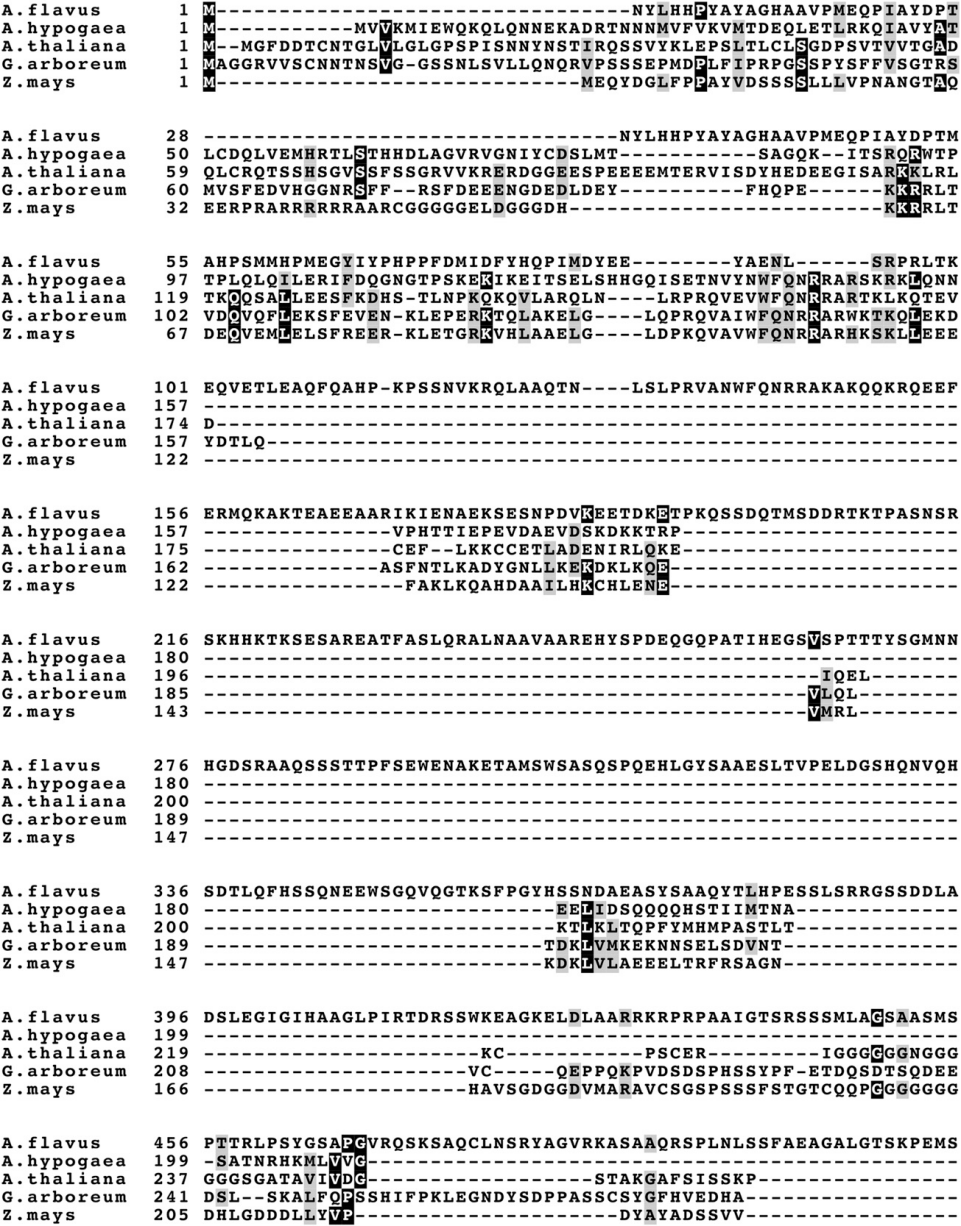
- Multiple sequence alignment of *Aspergillus flavus* Hbx1 and possible closest predicted proteins in selected plant species. BLASTp search was carried out to find possible homologs in selected plant species and with the best hits. A MAFFT Sequence Alignment (https://www.ebi.ac.uk/Tools/msa/mafft/) was performed to show homology of amino acid sequences.

To determine if the *hbx1* product is conserved among plants and thus a possible viable target for control of *A. flavus*, a BLASTp search was performed to identify potential homologs. The best BLASTp hit for Hbx1 from *Arachis hypogaea*, *Zea mays*, *Gossypium arboretum*, and *Arabidopsis thaliana* were chosen for comparison to the *A. flavus* protein. Among these hits the query coverage of the results was very low (9–18%) localizing only around the homeobox domain located in Hbx1. Amino acid sequences of all species were then run through a MAFFT multiple sequence alignment. The results of the alignment are visualized in Figure 1. This result, together with the low percentage values, indicates that Hbx1 from *Aspergillus* is not conserved in these common plant hosts.

### hbx1 is a global genetic regulator in A. flavus

**Figure 2:**
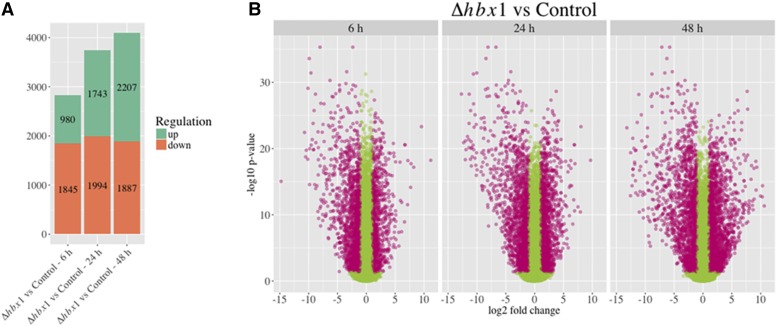
Number of genes influenced by *hbx1* A) Number of up-regulated (green) and down-regulated (orange) DEGs in Δhbx1 *vs.* control at the 6 h, 24 h, and 48 h time points. B) Volcano plot of log2 folder change *vs.* log10 P-value of all the genes in Δhbx1 *vs.* control at the 6 h, 24 h, and 48 h time points. DEGs are pink dots, other genes are shown as green dots. Pink dots with positive log2 fold change values are up-regulated DEGs. Pink dots with negative log2 fold change values are down-regulated DEGs. The x-axis represents the log2 of the fold change as determined by Limma. The y-axis is the log10 of the adjusted p value from Limma. The cut off-fold change value to determine differential expression is greater than 2 or less than 0.5. The cut off-adjusted p value to determine differential expression was greater than 0.05. Additional statistical representation of other comparison are in volcano plots located in Figure S1.

**Figure 3:**
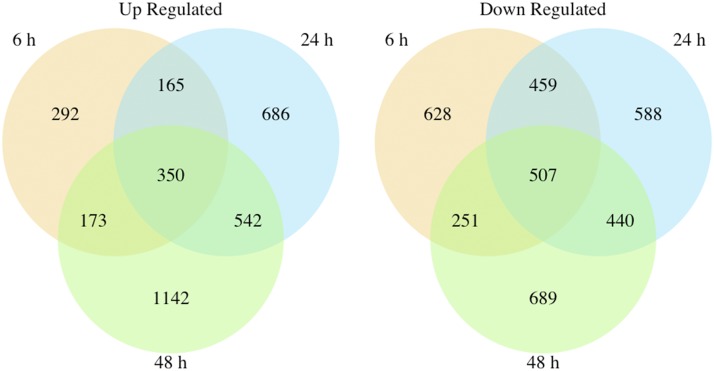
- Venn diagram visualizing the overlap of up regulated and down regulated genes in Δhbx1 *vs.* Control at the 6 h, 24 h, and 48 h time points.

Across the time points assessed, absence of *hbx1* caused a significant change in expression levels in more than 5000 genes in the *A. flavus* genome. Nearly 2000 genes were downregulated at each time point. In addition, while at the 6 h time point only 980 genes presented an increase in their expression, that number approximately doubled at the later time points (Figure 2, Table 1). Although a similar total amount of genes showed altered expression at each time point, there were not always the same DEGs at all three-time points. Only 350 genes of the entire genome were consistently upregulated by the loss of *hbx1*, while 507 genes experienced a significant decrease in expression at all three times points (Figure 3).

**Table 1:** – Percentage of *A.flavus hbx1-*dependent DEGs at each time point

		6 h	24 h	48 h	All 3 time points
Up regulated	Percent of total DEGs	16.17%	28.76%	36.42%	5.78%
	Percent of total genome	7.27%	12.93%	16.37%	2.60%
Down regulated	Percent of total DEGs	30.45%	32.90%	31.14%	8.37%
	Percent of total genome	13.68%	14.79%	13.99%	3.76%

### hbx1 is indispensable for normal secondary metabolism in A. flavus

**Figure 4:**
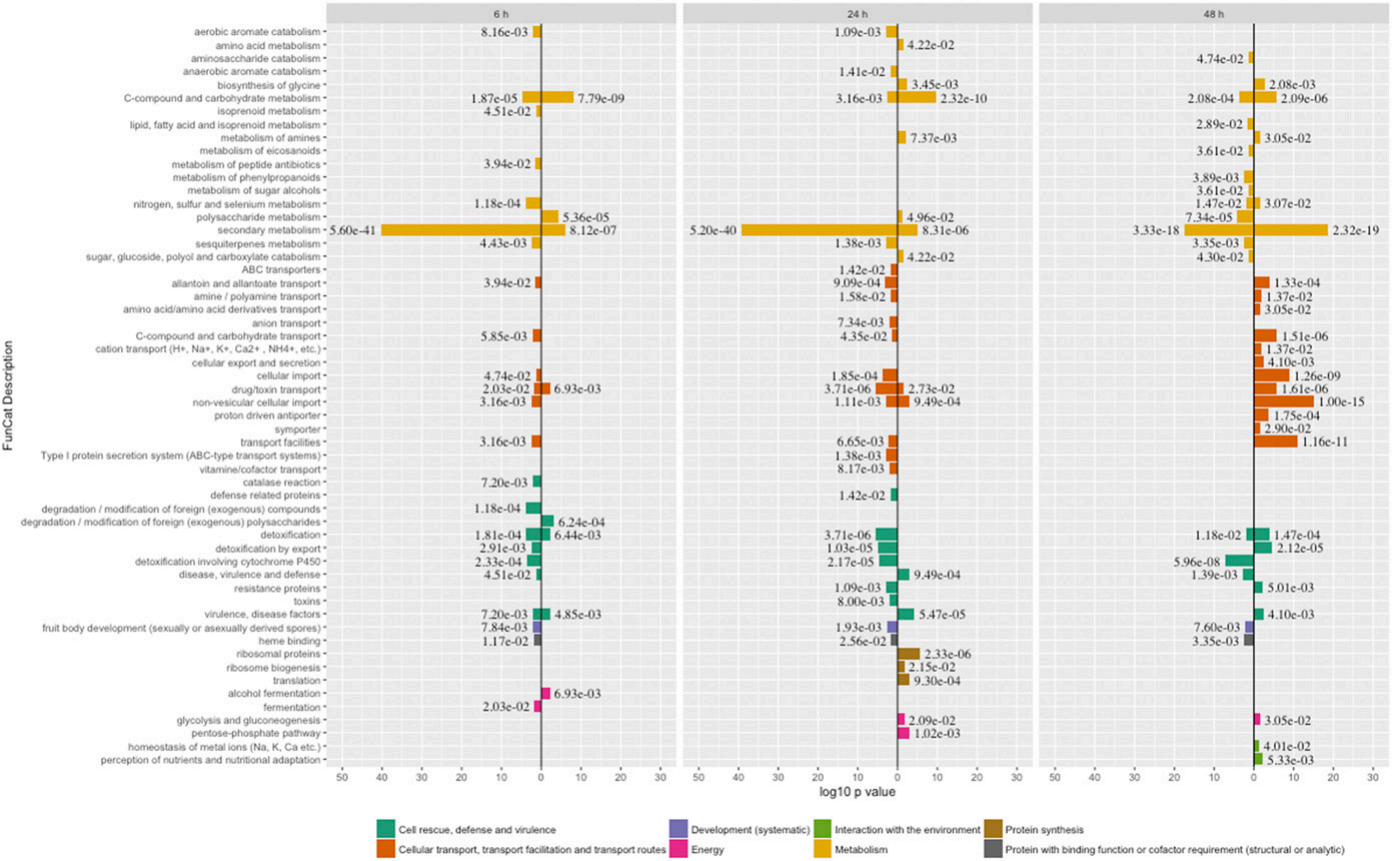
- FunCat terms associated with DEGs found in Δhbx1 *vs.* Control at 6 h, 24 h, and 48 h. The minus log10 of the p-value of DEGs in each term is proportional to the length of the bars. FunCat annotations and p-value as determined by FungiFun2(https://elbe.hki-jena.de/fungifun/fungifun.php): (i) metabolism is shown in orange, (ii) cellular transport, transport facilities and transport routes in light brown, (iii) cell rescue, defense & virulence in dark green, (iv) development in purple, (v) protein with binding function or cofactor requirement (structural or analytic) in black, (vi) protein synthesis in dark brown, (vii) energy in magenta, and (viii) interaction with the environment in light green. Down regulated genes are to the left of the origin and up regulated to the right.

To elucidate the regulatory scope of *hbx1* in *A. flavus*, a series of functional enrichment analyses were performed with the transcriptome data. Using the FungiFun2 platform we performed a Gene Ontology search using FunCat terms (Figure 4) (Priebe *et al.* 2014). The analysis revealed multiple enriched categories, with the largest one being related to metabolism, followed by cellular transport and cell rescue (the former particularly at 48 h) (Figure 4). Within this division of categories, metabolic genes involved in secondary metabolism were the largest group affected by loss of *hbx1*. At all-time points, most of the DEGs associated with secondary metabolism were downregulated in the absence of *hbx1*. GO terms were also used for functional analysis from FungiDB and are represented in Figure S2. This data also supports the pattern that secondary metabolism is the largest *hbx1*-affected category.

**Figure 5:**
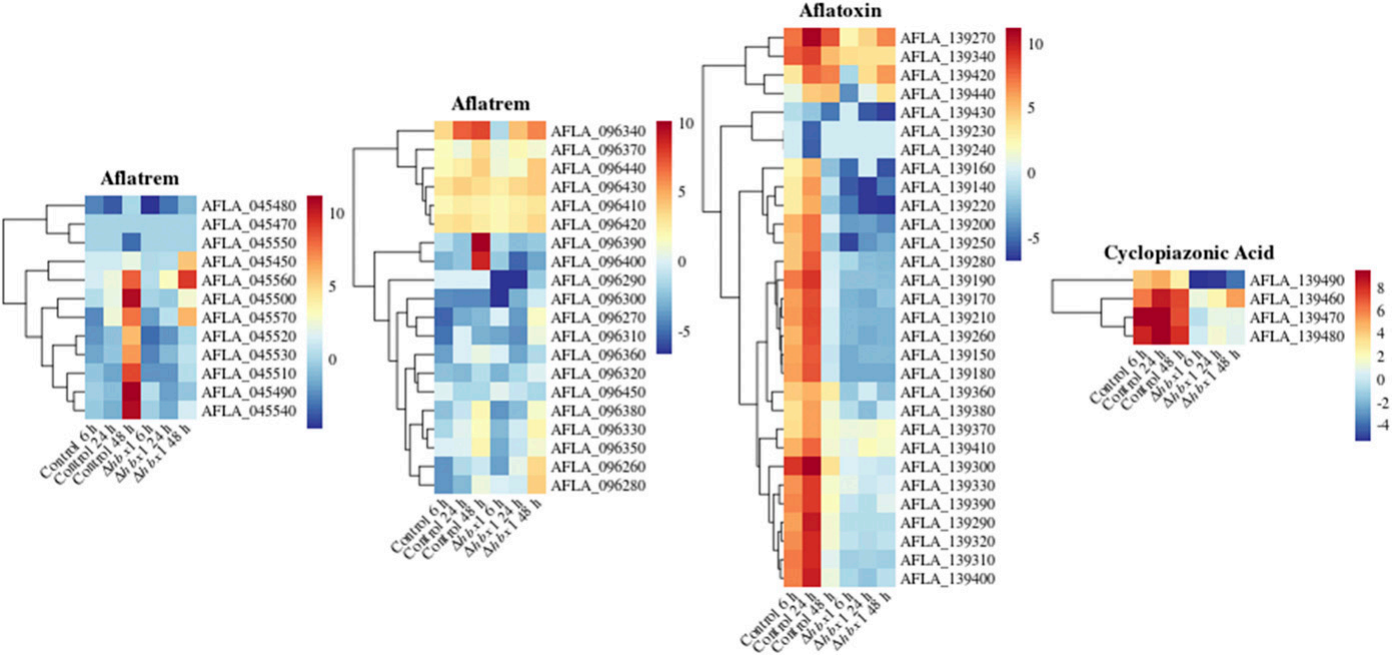
Heat map of RPKM values of genes on a log scale found in secondary metabolite gene clusters of aflatrem, aflatoxin, and cyclopiazonic acid (CPA). The RPKM value of each gene was calculated by averaging all the RPKM values of all replicates corresponding to that treatment at three different time points: 6 h, 24 h, and 48 h.

In a previous study we discovered that *hbx1* is a positive regulator of aflatoxin, aflatrem, and cyclopiazonic acid biosynthesis (Cary *et al.* 2017). The current transcriptome analysis provides further insight into *hbx1* regulation of biosynthetic gene clusters of those mycotoxins (Figure 5). In the aflatoxin cluster the majority of the genes are suppressed in the mutant strain. Previously mRNA transcripts from the aflatrem clusters were detected at approximately 48 h in the wild- type (Nicholson *et al.* 2009), coinciding with our observations (Figure 5), however such an increase was not observed in the *hbx1* deletion mutant. In addition, all the genes in the cyclopiazonic acid genes cluster were down regulated in the absence of *hbx1* (Figure 5).

**Figure 6:**
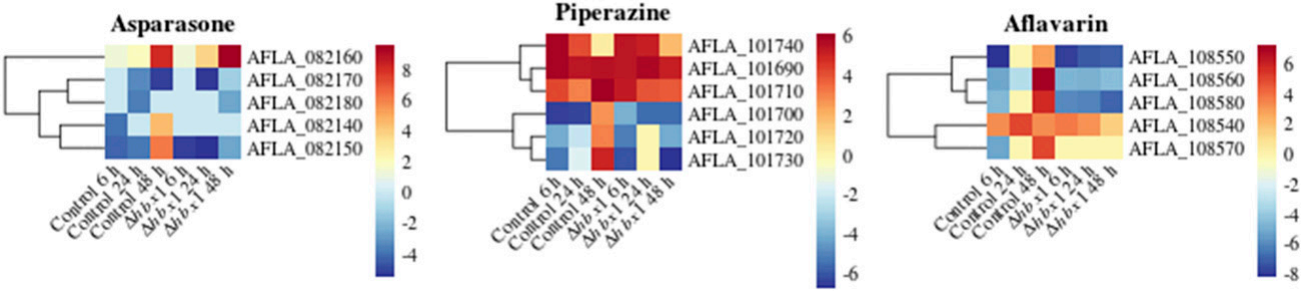
Heat map of RPKM values of genes on a log scale found in sclerotia related -secondary metabolite gene clusters of asparasone, piperazine, and aflavarin. The RPKM value of each gene was calculated by averaging all the RPKM values of all replicates corresponding to that treatment at three different time points: 6 h, 24 h, and 48 h.

Other gene clusters involved in the production of secondary metabolites known to be associated with sclerotial development were also affected, particularly at the last time point, such as the asparasone, piperazine, and aflavarin gene clusters (Figure 6). In addition, other secondary metabolite gene clusters in *A. flavus*, without a described associated product (orphan clusters) were shown to be regulated by *hbx1* (Figure S3). Other clusters were also affected but to a lesser extent (Table S1).

### hbx1 is a master regulator of developmental regulatory genes and other transcription factors in A. flavus

The *hbx1* gene is necessary for normal conidiation and sclerotial development in *A. flavus* (Cary *et al.*, 2017), and it was shown to affect the expression of key developmental regulators, such as *brlA*, the master regulator of the asexual development (conidia) in *Aspergillus*. In addition, *hbx1* also affected the expression of *veA*, which regulates multiple aspects of fungal development as well as influencing production of secondary metabolites (Calvo *et al.*, 2016). In our current study, a list of selected *A. flavus* developmental regulatory genes (experimentally characterized in *A. flavus* and/or in other fungi), was applied to our data analysis to better understand the mechanism of action of *hbx1* in *A. flavus* regulatory networks (Table S2). Our results revealed that several additional developmental transcription factors were also *hbx1*-dependent, such as the terminal gene in the central conidiation pathway, *wetA* (Wu *et al.*, 2017) the fluffy genes *flbA*, *flbC*, *flbD*, and *fluG* (Purschwitz *et al.*, 2008, Garzia *et al.*, 2010, & Kwon *et al.*, 2010, Chang *et al.* 2012), as well as the mating gene MAT1-1 (Ramirez-Prado *et al.*, 2008).

Our *hbx1* data analysis also extended to other *A. flavus* transcription factors. A list of *A. flavus* transcription factors were obtained from the Fungal Transcription Factor Database and used to search for DEGs. Annotations of the transcription factors were derived from NCBI. From these results, of the over six hundred transcription factors identified in this fungus, almost four hundred of them are influenced by *hbx1* (Table S3). A subset of well-characterized genes from that list are also represented in Table 2.

**Table 2:** Annotated *hbx1*-dependent transcription factors. A list of *A. flavus* transcription factors was obtained from the Fungal Transcription Factor Database and compared to the list of *hbx1* dependent DEGs. Annotations were retrieved from NCBI (full list is shown in Table S3). Expression values are those between the wild type (WT) and Δ*hbx1* at all time points assayed

Gene	AFLA ID	Description	6 h	24 h	48 h
*abp2*	AFLA_081210	ARS binding protein Abp2, putative	−0.02695	−1.05237	−0.83165
*aflO*	AFLA_139220	aflO/ omtB/ dmtA/ O-methyltransferase B	−8.01939	−10.9727	−2.94881
*aflP*	AFLA_139210	aflP/ omtA/ omt-1/ O-methyltransferase A	−9.18684	−11.8992	−2.50201
*aflR*	AFLA_139360	aflR / apa-2 / afl-2 / transcription activator	−6.93292	−5.75199	−5.3899
*amdA*	AFLA_048870	C2H2 transcription factor (AmdA), putative	−1.27305	0.369593	−0.66252
*amdR*	AFLA_028560	C6 transcription factor (AmdR), putative	−1.0392	−0.75555	−0.66391
*amdX*	AFLA_002290	C2H2 transcription factor (AmdX), putative	−1.02402	−1.62432	−0.7989
*amyR*	AFLA_026160	C6 transcription factor (AmyR), putative	0.718895	1.399027	1.196918
*areA*	AFLA_049870	GATA transcriptional activator AreA	−0.76928	−2.688	0.534173
*areB*	AFLA_136100	GATA transcription factor (AreB), putative	0.136046	−1.10235	0.413972
*azf1*	AFLA_054800	C2H2 transcription factor (Azf1), putative	−0.59027	−3.74734	−3.02994
*brlA*	AFLA_082850	C2H2 type conidiation transcription factor BrlA	0.391684	−2.17902	−2.37438
*cnjB*	AFLA_051900	zinc knuckle transcription factor (CnjB), putative	3.893041	0.290236	2.115113
*creA*	AFLA_134680	C2H2 transcription factor (Crea), putative	−0.50862	−0.56638	−1.0953
*ctf1B*	AFLA_012010	C6 transcription factor (Ctf1B), putative	−0.04607	0.620485	1.55645
*erg2*	AFLA_069460	C2H2 transcription factor (Egr2), putative	−1.55121	−0.57147	−1.05583
*flbC*	AFLA_137320	C2H2 conidiation transcription factor FlbC	−0.72449	−2.36931	−0.71241
*flbD*	AFLA_080170	MYB family conidiophore development protein FlbD, putative	−1.18991	−2.3671	−3.69073
*hpa3*	AFLA_131640	HLH transcription factor (Hpa3), putative	−0.97532	−1.5551	−2.37946
*MAT-α-1*	AFLA_103210	mating-type protein MAT alpha 1	0.301373	0.231333	3.258805
*mbf1*	AFLA_086430	coactivator bridging factor 1 (Mbf1), putative	0.637772	1.803474	0.237383
*nirA*	AFLA_093040	C6 transcription factor (NirA), putative	−0.36009	−1.05781	0.124633
*nosA*	AFLA_025720	C6 transcription factor NosA	−5.37861	−8.60563	−3.24851
*nsdD*	AFLA_020210	sexual development transcription factor NsdD	−0.29566	−1.18101	−0.97544
*pcaG*	AFLA_012100	NDT80_PhoG domain protein PcaG	−1.3881	−1.5446	−0.30405
*regA*	AFLA_073870	C6 transcription factor RegA, putative	−0.25045	0.20309	1.643673
*rfeC*	AFLA_044060	C2H2 transcription factor (RfeC), putative	−0.42645	−1.10049	0.597632
*rpn4*	AFLA_017640	C2H2 transcription factor (Rpn4), putative	0.491655	0.477596	3.176475
*seb1*	AFLA_110650	C2H2 transcription factor (Seb1), putative	−0.18746	−0.02585	1.021753
*sep1*	AFLA_048110	forkhead transcription factor (Sep1), putative	−0.65362	−1.43862	−0.5192
*snt2*	AFLA_029990	PHD finger and BAH domain protein (Snt2), putative	0.07615	−0.12486	1.163666
*srrA/skn7*	AFLA_034540	stress response transcription factor SrrA/Skn7, putative	−0.77849	−1.28158	0.119597
*ssb3*	AFLA_093820	ssDNA binding protein Ssb3, putative	−0.00201	0.813483	−1.02252
*steA*	AFLA_048650	sexual development transcription factor SteA	−0.53006	−1.08547	−0.48395
*stuA*	AFLA_046990	APSES transcription factor StuA	−1.62823	−2.5412	−1.79524
*swi5*	AFLA_031400	C2H2 transcription factor Swi5	−0.54244	−1.11596	−0.0182

### Prediction of hbx1-dependent genes possibly involved in virulence

Previously Dolezal *et al.* (2013) analyzed the transcriptome of *A. flavus* during the infection of maize kernels. This study compared the gene expression profile of *A. flavus* during infection of viable kernels to that of non-viable. The data from this study was assessed into two groups, genes that were upregulated during infection of viable kernels and those downregulated. We compared these groups of genes to the *hbx1*-dependent transcriptome from our study. In total, 1125, 1451, and 1672 genes were differentially expressed in both studies at the 6, 24, and 48 h, respectively (Table S4). Table S4 also shows genes that exhibited the same trend over three-time points as well as genes that presented an expression pattern opposite that depicted in the Dolezal *et al.* virulence study compared to *hbx1*-dependent DEGs. Among them, 75 genes were found upregulated during infection of viable seeds but downregulated in the *hbx1* mutant. Conversely 20 genes were found to present lower expression levels during infection while having increased expression in the *hbx1*mutant.

In the aforementioned study, genes encoding transcription factors, involved in secondary metabolism, as well as the fungal secretome were upregulated and predicted to be potential virulence factors (Dolezal *et al.*, 2013). We further compared differentially expressed secretory genes from that study to the *hbx1* transcriptome study. From this selected data set, 164, 196, and 235 secretory genes where differentially expressed in the absence of *hbx1* at 6, 24, and 48 h respectively (Table S5).

### Identification and visualization of gene regulatory networks correlated with hbx1 expression and knockout

**Figure 7:**
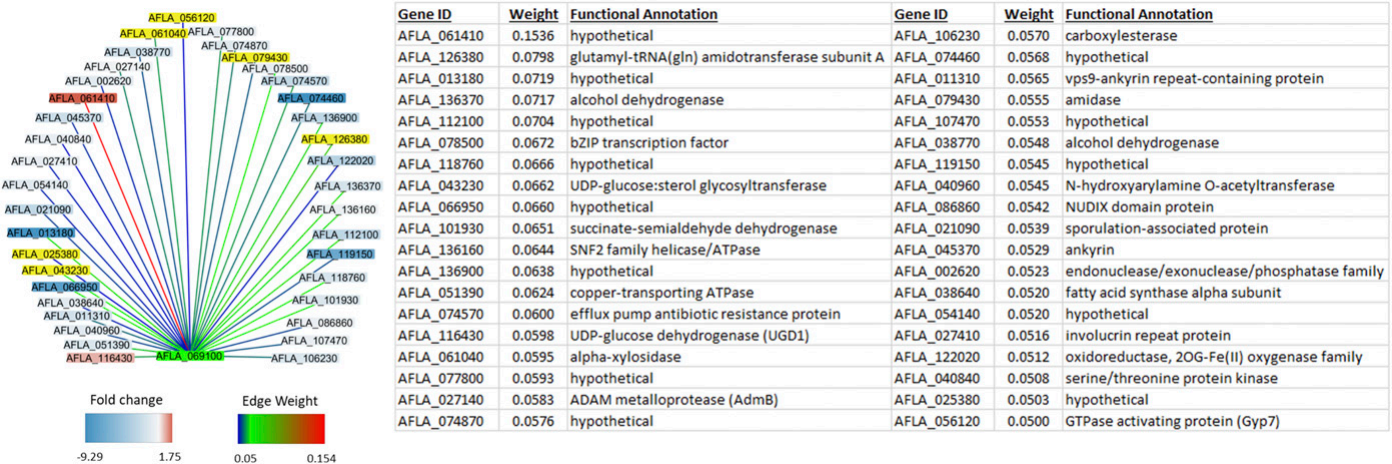
Weighted Gene Co-expression Network Analysis was conducted using read counts from the isogenic control strain to identify genes co-expressed with *hbx1* (AFLA_069100) and illustrated using Cytoscape (left). Edge coloration reflects the TOM value, indicating the relative significance of gene Co-expression (“Edge Weight”). The Node color reflects gene rlog2 fold changes in the 24 h sample of *hbx1* knockout mutant relative to control (“Fold change”). Yellow indicates no change in relative expression levels. The functional annotation from *Aspergillus flavus* strain 3357 (Accession: GCA_000006275.2) is indicated for each AFLA gene Identifier (right).

Expression values (variant stabilized read counts) from the RNA-seq data were subjected to WGCNA to identify networks of genes that are co-expressed with wild-type *hbx1*, and therefore potentially impacted by the *hbx1* deletion. The homeobox transcription factor showed highest co-expression correlation with a hypothetical protein (AFLA_061410), (Figure 7), which was significantly upregulated in the *hbx1* mutant (∼4 fold), and shared a high sequence similarity to a putative DNA methyltransferase. The putative hypothetical proteins down-regulated in the *hbx1* mutant (AFLA_013180 and AFLA_066950) share a sequence similarity with an S-adenosyl-methionine dependent methyltransferase and a flavin adenine dinucleotide -binding proteins, respectively. These putative methyltranferases highly associated with *hbx1* expression (and affected in the *hbx1* mutant) are excellent candidate regulatory genes for near-downstream regulation by *hbx1*.

## Discussion

Previous studies showed that development is genetically associated with secondary metabolism, (Calvo *et al.* 2002; Calvo and Cary 2015). Recent published work also demonstrated that the *hbx1* gene is one of those genetic links that is required for normal conidiation, sclerotial formation as well as secondary metabolism in the aflatoxin-producer and agriculturally important fungus *A. flavus* (Cary *et al.*, 2017). In the current study we showed that while homeobox domains similar to that present in the Hbx1 protein are found throughout various phyla, the rest of the *A. flavus* Hbx1 amino acid sequence is not conserved *in planta*. This suggests that *hbx1* could be a good target for strategies to control *A. flavus* infection that would not result in any off-target effects in agriculturally important crops susceptible to this opportunistic pathogen.

To gain insight into *A. flavus hbx1* mechanism of action we investigated the extent of its regulatory scope performing a transcriptome study using RNA sequencing. Our analyses revealed a broad effect of *hbx1* on the genome; in the absence of *hbx1* more than 20% of the *A. flavus* genome presents changes in expression. In addition, we observed that the number of genes governed by *hbx1* increases with time in the fungal culture. Based on our results, Hbx1 is a dynamic transcriptional regulator that, while it controls the expression of 857 genes at all time points assessed, a greater number of *hbx1*-dependent genes were affected at specific time points analyzed.

Our functional enrichment analysis indicated that while categories involved in cell rescue and defense, development and cellular transport were shown to be under the control of *hbx1*, the largest group of *hbx1*-dependent genes corresponds to the category of metabolism. Twelve secondary metabolite gene clusters, out of 56 clusters identified in *A. flavus*, including the kojic acid cluster (Ehrlich and Mack, 2014 & Ammar *et al.* 2017), were under *hbx1* control. Of these 12 clusters, five of them were not associated with a known metabolic product, however the remaining clusters have already been characterized. Four of these clusters have been shown to be involved in the synthesis of potent mycotoxins, aflatoxin, aflatrem (split into two clusters), and cyclopiazonic acid (Yu *et al.*, 2004, Chang *et al.*, 2009, Nicholson *et al.*, 2009). In addition, genes in the clusters involved in the production of asparasone, piperazine, and aflavarin were also shown to be suppressed in the absence of *hbx1*. These three metabolites are associated with sclerotial development (Calvo and Cary 2015). Both asparasone and aflavarin are specifically found within these structures (Cary *et al.* 2014, & Cary *et al.* 2015), and genes located in the piperazine cluster have been shown to affect their development (Forseth *et al.* 2012). Fungi concentrate secondary metabolites in reproductive structures for defense against herbivores and insects (Wicklow 1988, Gloer 1995, Gloer 1997, & Gloer 2007). Horn *et al.* (2009, 2014) reported ascospore-bearing ascocarps embedded within sclerotia of *A. flavus* and *A. parasiticus*. In these aflatoxin-producers, sclerotia play an important role as resting structures capable of surviving environmental extremes remaining viable after several years in the crop fields (Coley-Smith and Cooke 1971), and the *hbx1*-dependent secondary metabolites present in them contribute to their survival against biotic stress and possibly abiotic stresses. Since deletion of *hbx1* results in abolishment of sclerotia in the fungus, it is possible that the effect of *hbx1* on the expression of some of these secondary metabolite gene clusters specifically associated with a particular morphological structure could be indirect, by affecting developmental regulators that are repressed in the absence of *hbx1*. Whether the effect on these clusters is direct or indirect, these studies indicate an important role of *hbx1* in *A. flavus* survival, promoting the formation of resistant structures and a chemical arsenal critical for defense against microbes, predators and other environmental insults.

The *hbx1* gene is also necessary for conidiation. Our transcriptome analysis also indicated that the *brlA* central regulatory pathway is under *hbx1* control, not only affecting *brlA*, but also *wetA*, a developmental regulator conserved in *Aspergillus* species (Wu *et al.*, 2018). Furthermore, the aconidial phenotype of Δ*hbx1* resemble that of the fluffy mutants described in *A. nidulans* that revealed the *flb* regulatory pathway (reviewed by Ruger-Herreros *et al.* 2011; Krijgsheld *et al.* 2013). Indeed, *A. flavus flbA*, *flbC*, *flbD*, and *flbE* homologs (Chang *et al.*, 2012) are down regulated in the absence of *hbx1* while in the same strain at the early time point *fluG* had a significant increase of expression. This indicates that *hbx1* is a regulator of these conidiophore biogenesis genes, and expression of some of these genes over time is significantly different from that observed in the control strain.

It is possible that Hbx1 might not bind directly to the promoters of the central regulatory pathway genes but affects their expression by controlling expression of genes upstream in the regulatory hierarchy. Examples of these might be genes like *ppoC* and *stuA*. Both of these genes have been shown to affect conidiophore development via *brlA* (Dutton *et al.*, 1997, Tsitsigiannis *et al.*, 2004, & Sheppard *et al.*, 2005). In addition, other developmental genes were also under the influence of *hbx1*, for instance, the spore hydrophin gene *rodA* (Carrion *et al.*, 2013) and also the *nosA* gene, encoding a putative Zn(II)(2)Cys(6) transcription factor previously described in several *Aspergillus* species (Vienken and Fischer 2006, Soukup *et al.*, 2012, Zhao *et al.*, 2017). In *A. nidulans*, *nosA* is necessary for cleistothecial primordium maturation (Vienken and Fischer 2006), and its homolog in *A. flavus* has been reported to be required for sclerotial production (Zhao *et al.*, 2017). It is likely that the reduction in the expression of *nosA* in the *A. flavus hbx1* mutant could contribute to prevent sclerotial formation in this strain.

Since *hbx1* has a broad effect on *A. flavus* development and metabolism, we also investigated possible connections between *hbx1* and virulence during corn infection based on the previous report by Dolezal *et al.* (2013). In our study, the DEGs identified from the *hbx1*-dependent transcriptome were compared to those identified by Dolezal *et al.* (2013) in viable and non-viable infected corn kernels. This allowed us to predict genes possibly involved in virulence that are controlled by *hbx1*. Genes identified as upregulated in the corn infection study but suppressed in the Δ*hbx1* transcriptome study could potentially be involved in virulence. Approximately 300 DEGs were identified at all time points that fit this description. Among them is the *pes1* gene (AFLA_069330), that in *Aspergillus fumigatus* was found indispensable for virulence in the *Galleria mellonella* model (Reeves *et al.*, 2006). Other genes in this group were shown to affect spore germination and secondary metabolism, such as *sfk1*. In *Penicillium roqueforti*, silencing of *sfk1* alters condial germination and prevents production of roquefortine C, andrastin A, and mycophenolic acid (Torrent *et al.*, 2017). Out of the mentioned group of 300 DEGs, 75 were consistently suppressed in the Δ*hbx1* mutant (Table S4). In this subgroup, beyond genes located in the aflatoxin gene cluster, most of these genes have not been investigated, and could be potential genes of interest in future studies to identify *A. flavus* virulence factors.

Secreted proteins such as hydrolytic enzymes are essential for successful infection of the host by the fungus (Lo Presti *et al.*, 2015 -de Jonge *et al.*, 2011 & Kale and Tyler 2011). With this in mind, we focused on analyzing components of the secretome regulated by *hbx1*, specifically those genes that may play a role in virulence. FunSecKB2 analysis revealed that among the genes in the Dolezal *et al.* (2013) study, approximately 50 secretome-related genes were upregulated during infection of viable seeds, while those same genes were downregulated in our transcriptome study of the *hbx1* deletion mutant, at least at one time point, suggesting that this set of *hbx1*-dependent genes could be potentially be involved in virulence, for example genes encoding proteases (*i.e.*AFLA_057670), amylases (*i.e.*, AFLA_123170) and other hydrolases (*i.e.*, AFLA_065010, AFLA_088610, and AFLA_125970).

Weighted gene co-expression network analysis has been used to identify novel gene interactions by determining patterns of co-expression among several biological samples, which infers a functional relationship between genes. This process has been used to analyze RNA-seq data from *Aspergillus* species (Baltussen *et al.* 2018, Korani *et al.* 2018), and functional studies have demonstrated the validity of WGCNA (Calabrese *et al.*, 2017, Wang *et al.* 2017). Here we identify the network of co-expressed genes using the isogenic control strain and identified several genes that are both significantly co-expressed with *hbx1* and show altered expression patterns in the *hbx1* knockout mutant. Three genes of particular interest that demonstrated altered regulation and relatively high correlation values are annotated as hypothetical proteins (AFLA_ 013180, AFLA_061410, and AFLA_066950). The impact of these hypothetical genes on *A. flavus* biology will be the focus of future studies of *hbx1*-dependent gene regulation.

We demonstrated that the expression of thousands of genes is affected by *hbx1*. In addition, we showed that *hbx1*-dependent regulation in *A. flavus* is dynamic in a time-dependent manner. The *hbx1* gene is required for the production of structures needed for dissemination and survival of *A. flavus* and the production of detrimental secondary metabolites. This, together with the fact that Hbx1 is not conserved in other phyla suggest that this global regulator could be a target to develop novel methodologies to control the adverse health and economic impacts due to infection and aflatoxin contamination of many important crops by *A. flavus*.
